# Molecular Mechanisms of the Regulation of Liver Cytochrome P450 by Brain NMDA Receptors and via the Neuroendocrine Pathway—A Significance for New Psychotropic Therapies

**DOI:** 10.3390/ijms242316840

**Published:** 2023-11-28

**Authors:** Renata Pukło, Ewa Bromek, Anna Haduch, Agnieszka Basińska-Ziobroń, Wojciech Kuban, Władysława A. Daniel

**Affiliations:** Department of Pharmacokinetics and Drug Metabolism, Maj Institute of Pharmacology, Polish Academy of Sciences, Smętna 12, 31-343 Kraków, Poland; bartyzel@if-pan.krakow.pl (R.P.); bromek@if-pan.krakow.pl (E.B.); haduch@if-pan.krakow.pl (A.H.); ziobron@if-pan.krakow.pl (A.B.-Z.); kuban@if-pan.krakow.pl (W.K.)

**Keywords:** CP-101,606, activity/expression, liver, cytochrome P450, NMDA receptor, neuroendocrine regulation

## Abstract

Recent investigations have highlighted the potential utility of the selective antagonist of the NMDA receptor GluN2B subunit for addressing major depressive disorders. Our previous study showed that the systemic administration of the antagonist of the GluN2B subunit of the NMDA receptor, the compound CP-101,606, affected liver cytochrome P450 expression and activity. To discern between the central and peripheral mechanisms of enzyme regulation, our current study aimed to explore whether the intracerebral administration of CP-101,606 could impact cytochrome P450. The injection of CP-101,606 to brain lateral ventricles (6, 15, or 30 µg/brain) exerted dose-dependent effects on liver cytochrome P450 enzymes and hypothalamic or pituitary hormones. The lowest dose led to an increase in the activity, protein, and mRNA level of CYP2C11 compared to the control. The activities of CYP2A, CYP2B, CYP2C11, CYP2C6, CYP2D, and protein levels of CYP2B, CYP2C11 were enhanced compared to the highest dose. Moreover, CP-101,606 increased the CYP1A protein level coupled with elevated *CYP1A1* and *CYP1A2* mRNA levels, but not activity. The antagonist decreased the pituitary somatostatin level and increased the serum growth hormone concentration after the lowest dose, while independently decreasing the serum corticosterone concentration of the dose. The findings presented here unveil a novel physiological regulatory mechanism whereby the brain glutamatergic system, via the NMDA receptor, influences liver cytochrome P450. This regulatory process appears to involve the endocrine system. These results may have practical applications in predicting alterations in cytochrome P450 activity and endogenous metabolism, and potential metabolic drug–drug interactions elicited by drugs that cross the blood–brain barrier and affect NMDA receptors.

## 1. Introduction

Various factors, including diet, xenobiotics, and diseases, have the potential to influence cytochrome P450 activity. However, a comprehensive understanding of the physiological regulation of cytochrome P450 is crucial, as it can provide valuable insights into the influence of these various factors. Initially, research on the physiological regulation of cytochrome P450 primarily focused on liver-based mechanisms, considering the involvement of hormones and exogenous ligands in activating the cytoplasmic/nuclear receptors responsible for cytochrome P450 gene expression [1,2,3]. Nevertheless, the release of hormones that modulate cytochrome P450 genes is dependent upon the functioning of the nervous system [4,5,6]. The influence of the catecholaminergic [7,8,9] and serotonergic systems [10] has already been investigated. Nonetheless, the contribution of the brain glutamatergic system to this particular context remains inadequately elucidated and requires further investigation, since new psychotropic therapies involving that neurotransmitter system are emerging.

The hypothalamus, serving as a vital connection between the nervous and endocrine systems, receives significant innervation from the glutamatergic system, where signals are conveyed through various glutamate receptors, encompassing ionotropic (NMDA, AMPA, and KA receptors) and metabotropic (group I, II, and III mGlu receptors) receptors [11,12,13,14,15]. The hypothalamus receives both intrahypothalamic and extrahypothalamic glutamatergic innervation, the latter arising from the forebrain and the brainstem [16,17,18]. There is a dense glutamatergic innervation of growth hormone-releasing hormone (GHRH) containing neurons in the hypothalamic arcuate nuclei (ARC), as well as glutamatergic impact on corticotropin-releasing hormone (CRH), thyrotropin-releasing hormone (TRH) and somatostatin-synthesizing neurons in the hypothalamic paraventricular nuclei (PVN) [11,12,16,19]. Thus, the brain glutamatergic system can influence the expression of hepatic cytochrome P450 by affecting the hypothalamic parvocellular system and subsequently by modulating the hormone secretion from the anterior pituitary [11,20,21,22,23,24,25]. Moreover, the pharmacological impact of drugs acting on glutamate receptors extends to the liver, where these receptors are also expressed [26,27,28]. This issue bears considerable importance, especially in light of the expanding research on novel pharmaceuticals targeting glutamate receptors, such as the selective NMDA receptor antagonists utilized for managing depression [29,30,31,32].

Our recent investigations involving male rats were subjected to intraperitoneal treatment with CP-101,606, a selective antagonist targeting the GluN2B subunit of NMDA receptors, revealed potential interactions between the brain glutamatergic system and endocrine mechanisms, which influenced the expression and activity of cytochrome P450 [33]. In particular, the 5-day administration of CP-101,606 resulted in decreased CYP1A, CYP2A, CYP2B, CYP2C11, and CYP3A activities, while CYP2C6 activity (less susceptible to hormonal regulation) remained unaffected. These changes in enzymatic activity were accompanied by corresponding alterations in CYP protein and mRNA levels. Furthermore, a concurrent reduction in the pituitary growth hormone-releasing hormone level, as well as the serum growth hormone and corticosterone concentrations were observed.

However, it is crucial to elucidate the potential influence of the drug on cytochrome P450 regulation at the hepatic level and its direct effect on the *CYP* gene expression or enzyme protein function. The observed outcomes of the systemic administration of CP-101,606 on the enzyme might arise from complex interactions between the drug and CYP enzymes, both direct (at the level of hepatocyte) and indirect (*via* the neuroendocrine system), which makes it challenging to determine the exclusive role and mechanisms of the central neuroendocrine regulation of CYP enzymes’ expression in the liver. To address concerns about the possible peripheral drug–CYP interactions, in our current work, we investigated the liver CYP enzyme expression and activity after the intracerebral injection of CP-101,606 to avoid any direct action of the compound at the level of hepatocyte. The obtained results are compared with those obtained after the intraperitoneal administration of CP-101,606.

## 2. Results

### 2.1. The Impact of Intracerebral Administration of CP-101,606 on the Hepatic Activity of CYP1A, CYP2A, CYP2B, CYP2C11, CYP3A, CYP2C6, and CYP2D Enzymes

**Figure 1:**
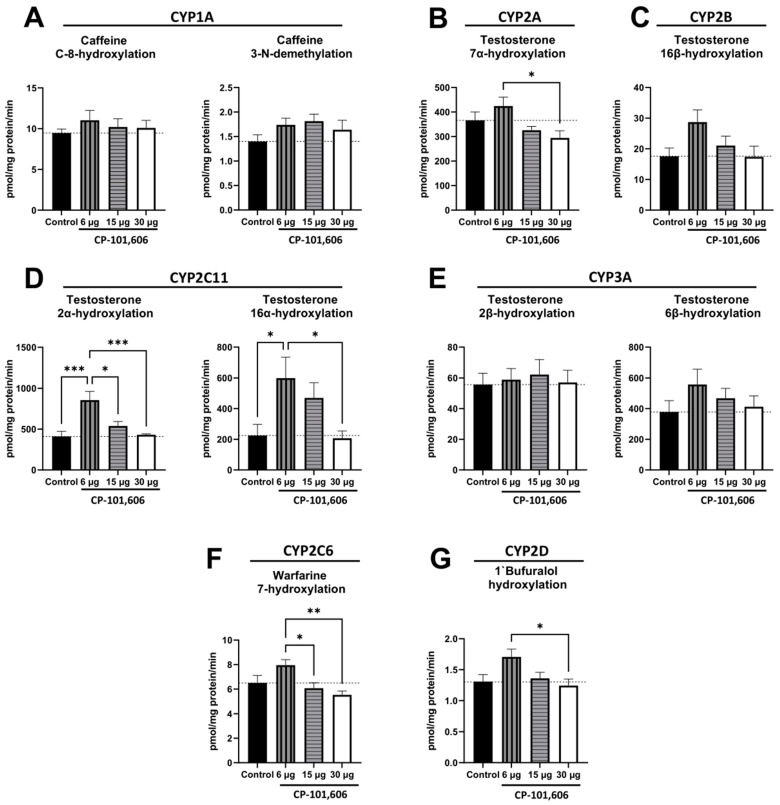
The impact of a 5-day intracerebral administration of CP-101,606, a selective antagonist targeting the GluN2B subunit of the NMDA receptor, at three different doses (6, 15, or 30 µg/brain) on cytochrome P450 enzyme activity. The activity of various CYP enzymes was assessed as follows: CYP1A, measured by the rates of caffeine 8-hydroxylation and 3-N-demethylation (**A**); CYP2A, measured by the rates of testosterone 7α-hydroxylation (**B**); CYP2B, measured by the rates of testosterone 16β-hydroxylation (**C**); CYP2C11, measured by the rates of testosterone 2α- and 16α-hydroxylation (**D**); CYP3A, measured by the rates of testosterone 2β- and 6β-hydroxylation (**E**); CYP2C6, measured by the rates of warfarin 7-hydroxylation (**F**); and CYP2D, measured by the rates of bufuralol 1′-hydroxylation (**G**). The values are presented as the mean ± S.E.M. (n = 9–10 rats). Statistical analysis was performed using one-way ANOVA followed by Tukey’s *post hoc* test. Statistical significance was indicated as follows: * *p* < 0.05; ** *p* < 0.01; *** *p* < 0.001.

The impact of the five-day intracerebral administration of CP-101,606 in three different doses (6, 15, or 30 µg/brain) on the enzymatic activity of cytochrome P450 enzymes in rat liver microsomes was investigated (Figure 1A–G). The effects of CP-101,606 were found to be dose-dependent. At the lowest dose, CP-101,606 increased the activity of CYP2C11, assessed by the rate of testosterone 2α- and 16α-hydroxylation, compared to both the control group and the higher antagonist dose (Figure 1D).

The lowest dose of CP-101,606 also led to increased activities of CYP2C6 (Figure 1F), assessed by warfarin 7-hydroxylation, and CYP2D (Figure 1G), assessed by bufuralol 1′-hydroxylation, compared to both the control group and to the higher antagonist dose. Moreover, the activity of CYP2A (Figure 1B), assessed by testosterone 7α-hydroxylation, was enhanced after the lowest dose compared to the highest dose. However, CP-101,606 did not exhibit any significant effects on the activity of CYP1A (Figure 1A), CYP2B (Figure 1C), and CYP3A enzymes (Figure 1E), as assessed by the caffeine C-8-hydroxylation and 3-N-demethylation, testosterone 16β-hydroxylation, and testosterone 2β- and 6β-hydroxylation, respectively. However, tendencies to increase the activity of CYP2B (16β-hydroxylation) and CYP3A (6β-hydroxylation) after the lowest dose of CP-101,606 were noticed. Based on the above results concerning CYP enzyme activity, the lowest and highest doses of CYP-101,606 were chosen for further molecular studies.

### 2.2. The Impact of the Intracerebral Administration of CP-101,606 on the Protein Levels of CYP1A, CYP2A, CYP2B, CYP2C11, CYP3A, CYP2C6, and CYP2D in the Liver

**Figure 2:**
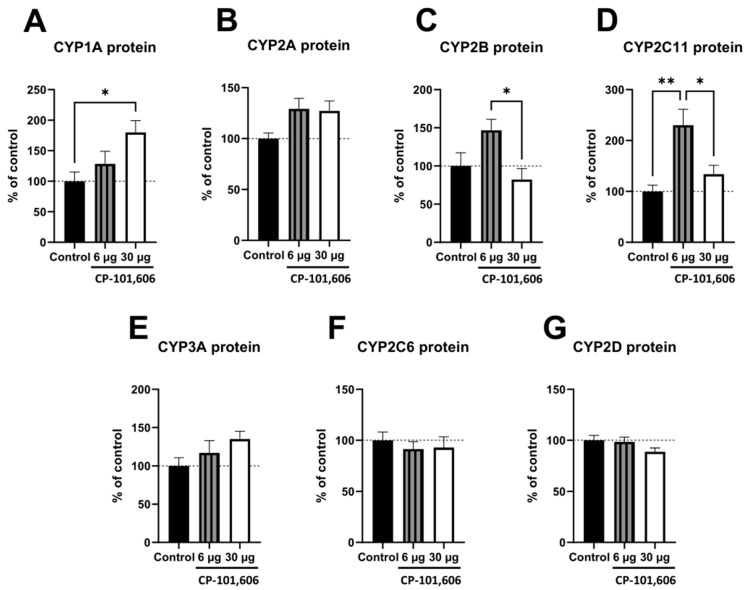
The influence of a 5-day intracerebral administration of CP-101,606, a selective antagonist targeting the GluN2B subunit of the NMDA receptor, at two selected doses (6 or 30 µg/brain) on the protein levels of cytochrome P450 enzymes. The following CYP enzymes were analyzed: CYP1A (**A**), CYP2A (**B**), CYP2B (**C**), CYP2C11 (**D**), CYP3A (**E**), CYP2C6 (**F**), and CYP2D (**G**). The values are expressed as the mean ± S.E.M. (n = 8 rats). One-way ANOVA followed by Tukey’s *post hoc* test was utilized for statistical analysis. Statistical significance was represented as follows: * *p* < 0.05; ** *p* < 0.01.

Western blot analysis was used to evaluate the protein levels of CYP enzymes in the liver microsomes of the control group and two selected doses of CP-101,606 (6 and 30 µg/brain) (Figure 2A–G). Changes in the protein levels of CYP2C11 matched the changes in its activity pattern, where the lowest dose of CP-101,606 resulted in an increase in the protein level compared to both the control and the highest antagonist dose (Figure 2D).

**Figure 3:**
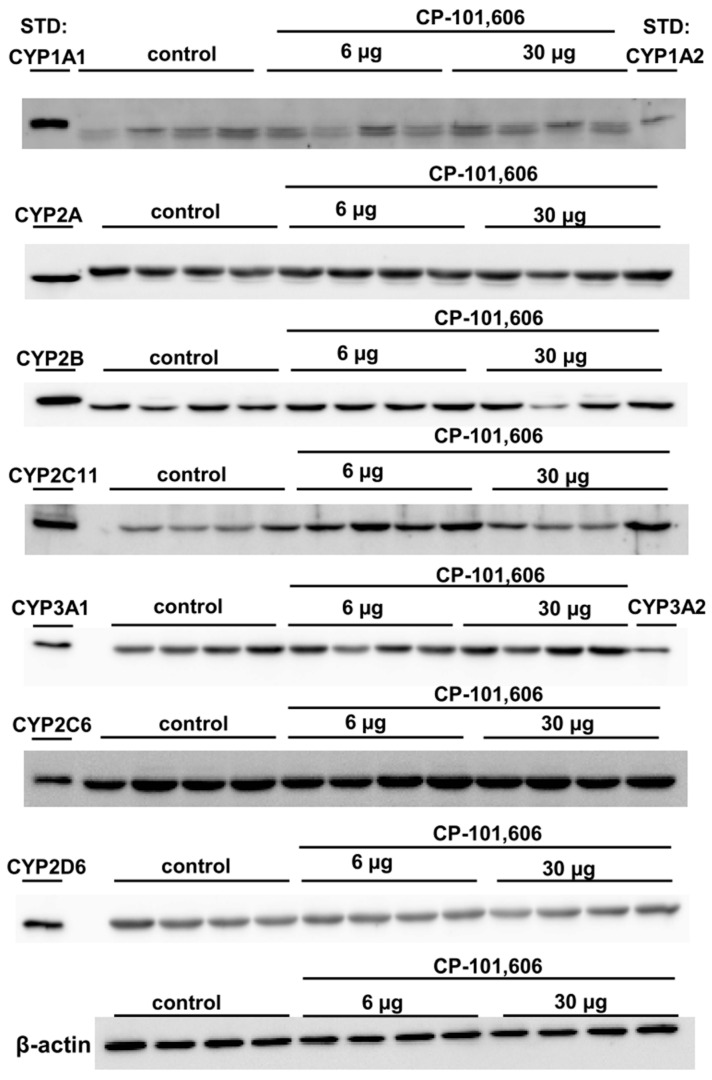
The impact of a 5-day treatment with two selected doses (6 or 30 µg/brain) of CP-101,606 on the intensity of cytochrome P450 protein bands (CYP1A, CYP2A, CYP2B, CYP2C11, CYP3A, CYP2C6, and CYP2D) in liver microsomes. Rat cDNA-expressed CYP enzymes were utilized as standards in the analysis. The figure shows the Western immunoblot analysis of representative CYP protein bands. The mean values ± S.E.M. (n = 8 rats) of the respective CYP protein levels are presented in Figure 2A–G. STD—standard.

Additionally, a significant increase in the CYP1A protein levels (Figure 2A) was observed at the highest dose of the antagonist in comparison to the control group (without corresponding activity changes). Similarly, an increase in the CYP2B protein levels (Figure 2C) was found at the lowest dose of the antagonist relative to the highest dose (with a corresponding increasing tendency in the enzyme activity). However, no significant changes in protein levels were observed for CYP2A, CYP2C6, CYP2D, and CYP3A enzymes (Figure 2B,E–G). Figure 3 displays the representative CYP protein bands resulting from Western immunoblot analysis in rats treated with CP-101,606.

### 2.3. The Impact of Intracerebral Administration of CP-101,606 on the Hepatic mRNA Levels of CYP1A1, CYP1A2, CYP2B1, CYP2B2, and CYP2C11

**Figure 4:**
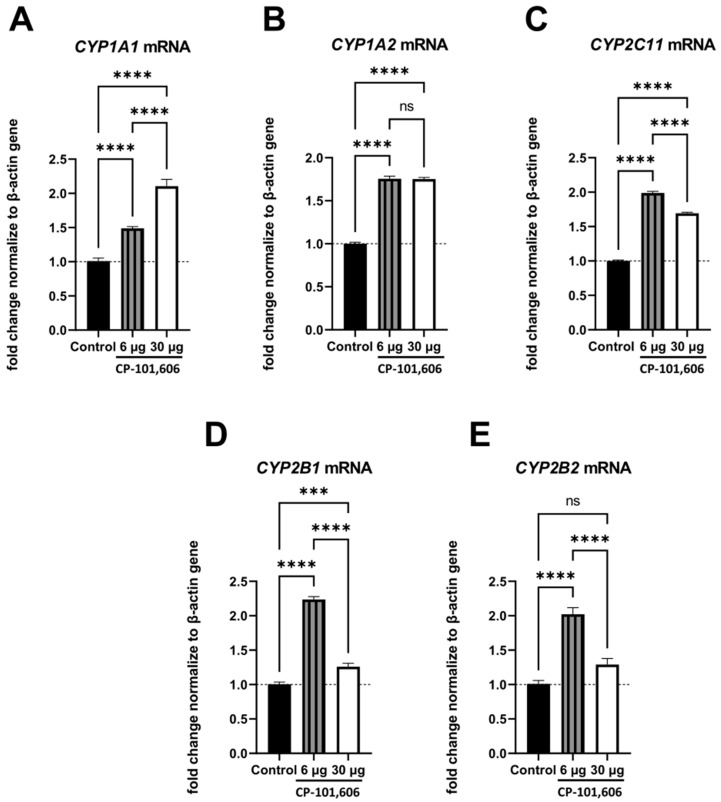
The influence of a 5-day intracerebral administration of CP-101,606, a selective antagonist targeting the GluN2B subunit of the NMDA receptor, at two selected doses (6 or 30 µg/brain) on the mRNA levels of the cytochrome P450 enzymes: *CYP1A1* (**A**), *CYP1A2* (**B**), *CYP2C11* (**C**), *CYP2B1* (**D**), and *CYP2B2* (**E**). The data are presented as the mean ± S.E.M. (n = 9–10 rats). One-way ANOVA followed by Tukey’s *post hoc* test was employed for statistical analysis. Statistical significance was indicated as follows: *** *p* < 0.001; **** *p* < 0.0001; ns—non-significant.

The *CYP1A1*, *CYP1A2*, *CYP2B1*, *CYP2B2,* and *CYP2C11* genes, whose protein products were significantly changed, were chosen for assessing mRNA levels through the qRT-PCR method (Figure 4A–E). The comparison was made between the control group and the two groups treated with the lowest or highest doses of CP-101,606 (6 and 30 µg/brain).

The alterations in the *CYP2C11* mRNA level were consistent with the observed changes in protein level and activity (Figure 4E). The administration of the lowest dose of CP-101,606 led to a statistically significant increase in the *CYP2C11* mRNA level compared to both the control group and the group treated with the highest dose of the antagonist. Moreover, the highest dose of the antagonist resulted in a statistically significant increase in the mRNA level relative to the control group.

Changes in the mRNA levels of *CYP1A1* and *CYP1A2* were in accordance with those obtained in the protein level. In both cases, the lowest and the highest doses of CP-101,606 led to a statistically significant increase in the mRNA levels compared to the control group. Moreover, a significant increase observed in the *CYP1A1* mRNA level at the lowest dose of the antagonist was notably lower than that observed at the highest dose (Figure 4A). But unlike *CYP1A1*, no significant difference was observed in the *CYP1A2* mRNA levels between the groups treated with the lowest and highest doses of the antagonist (Figure 4B).

The lowest dose of CP-101,606 evoked a distinct increase in the *CYP2B1* and *CYP2B2* mRNA levels, while the highest dose only led to a subtle elevation in those mRNAs (Figure 4D,E).

### 2.4. The Influence of CP-101,606 Intracerebral Administration on the Levels of Hormones

**Figure 5:**
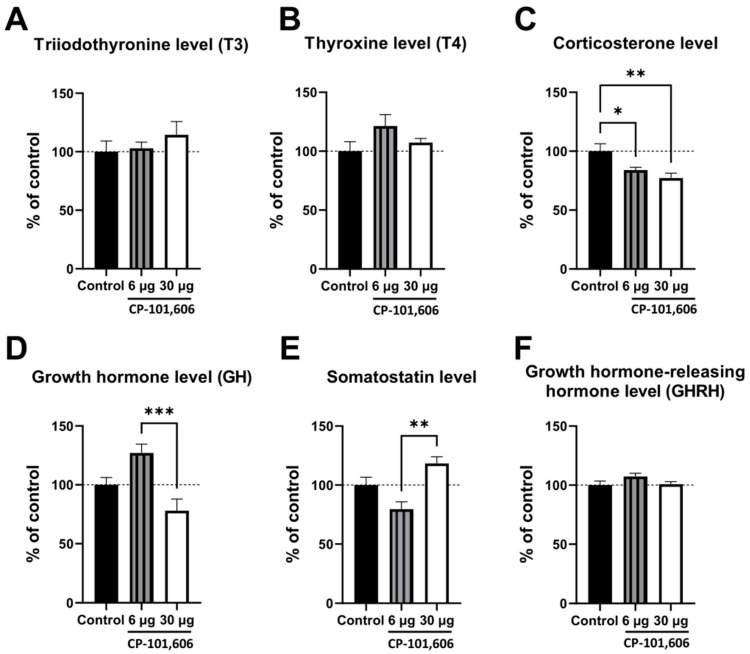
The influence of a 5-day intracerebral administration of CP-101,606, a selective antagonist targeting the GluN2B subunit of the NMDA receptor, at two selected doses (6 or 30 µg/brain) on the serum hormones: triiodothyronine (T_3_) (**A**), thyroxine (T_4_) (**B**), corticosterone (**C**), growth hormone (GH) (**D**), and pituitary gland hormones: somatostatin (**E**) and growth hormone-releasing hormone (GHRH) (**F**). The data represent the mean ± S.E.M. (8–10 rats). One-way ANOVA followed by Tukey’s *post hoc* test was used for statistical analysis. Statistical significance was denoted as follows: * *p* < 0.05; ** *p* < 0.01; *** *p* < 0.001.

The five-day intracerebral administration of CP-101,606 did not elicit any effects on thyroid hormone levels (T_3_, T_4_) (Figure 5A,B). However, there was a significant decrease in the corticosterone levels (Figure 5C) produced by both the lowest and the highest doses of CP-101,606. The lowest dose of the antagonist tended to increase the serum growth hormone concentration (Figure 5D) and to decrease the pituitary somatostatin level (Figure 5E).

In contrast, the highest dose of the antagonist tended to diminish the serum growth hormone concentration (Figure 5D) and to increase the pituitary somatostatin level (Figure 5E). Consequently, there was a significant difference in the hormone levels (GH, somatostatin) between the treatment with the lowest and highest dose (Figure 5D,E). No changes were observed in the level of pituitary growth hormone-releasing hormone (GHRH) (Figure 5F).

## 3. Discussion

The potential influence of the brain glutamatergic system on the central neuroendocrine regulation of cytochrome P450 expression has not been explored in depth to date. Our previous studies were carried out after the intraperitoneal, repeated administration of CP-101,606, a selective antagonist targeting the GluN2B subunit of NMDA receptors, showing a significant decrease in the cytochrome P450 expression and/or the activity accompanied by appropriate alterations in pituitary and serum hormones; however, the contribution of the peripheral action of the applied NMDA antagonist in the observed effects in liver CYPs could not be excluded [33].

Our present investigation was performed after the intracerebral administration of CP-101,606, indicating that the brain glutamatergic system plays a significant role in modulating cytochrome P450 expression and activity through central NMDA receptors and endocrine pathways. The intracerebroventricular and repeated injection of the antagonist led to alterations in the functioning of the hepatic cytochrome P450 enzymes in rats, as outlined in Table 1. The applied intracerebroventricular doses of CP-101,606 (6–30 µg icv.) were far lower than the pharmacological peripheral doses of the drug (20 mg.kg. ip.), which fully occupy NMDA receptors in the brains of rodents [33]. Therefore, we assume that a peripheral effect of CP-101,606 was negligible after intracerebral administration in our experiment.

The injection of CP-101,606 into brain lateral ventricles exerted dose-dependent effects (6–30 µg/brain) on the liver cytochrome P450 enzyme expression and activity and on pituitary or serum hormone levels. The lowest dose led to an increase in the expression and activity of CYP2C11 compared to the control, and such a tendency was observed for the CYP2B subfamily enzymes. Comparing the lowest and highest doses of CP-101,606, it is also noteworthy that the activities of CYP2A, CYP2B, CYP2C11, CYP2C6, and CYP2D as well as the protein levels of CYP2B and CYP2C11 observed after the lowest dose were enhanced compared to the highest dose. Moreover, CP-101,606 increased the CYP1A protein level coupled with the elevated *CYP1A1* and *CYP1A2* mRNA levels, but it did not change the enzyme activity. This suggests that other mechanisms, potentially post-translational modifications, may be involved in the regulation of CYP1A. The above changes in the CYP enzymes’ expression and activity were accompanied by changes in the serum growth hormone and the corticosterone concentrations and pituitary somatostatin levels. Interestingly, no significant alterations in the CYP3A enzymes were observed in spite of the fact that they share regulatory mechanisms with other CYP enzymes. It is noteworthy that the human counterpart enzymes CYP1A2, CYP3A4, CYP2Cs, and CYP2D6 are the main CYP enzymes involved in drug metabolism.

The studied cytochrome P450 enzymes are subjected to regulation by various hormones, including the growth hormone (GH), glucocorticoids (corticosterone), and thyroid hormones (triiodothyronine T_3_ and thyroxine T_4_). These hormones exert their regulatory effects through the activation of a specific membrane, cytoplasmic or nuclear receptors, thereby influencing the transcription of *CYP* genes [4,34,35,36,37,38]. Scientific investigations have revealed that the activation of NMDA receptors can lead to the stimulation of growth hormone (GH) secretion. However, it is important to note that this stimulus does not originate from the pituitary gland, but rather is generated at the level of GHRH and/or somatostatin neurons [23]. GH serves as the primary positive regulator of CYP2C11 and is also involved in regulating other CYP enzymes [34]. In accordance with the neuroendocrine pathway mentioned above, our experimental findings suggested that a 5-day intracerebral treatment with the lowest dose of the selective NMDA receptor GluN2B subunit antagonist CP-101,606 led to a diminution in the pituitary somatostatin level and consistently to an increase in serum GH concentration (with no change in the GHRH levels), which resulted in the enhancement of the GH-related expression (mRNA and protein) and activity of CYP2C11 in the liver. However, after the treatment with the highest dose of the antagonist, the CYP2C11 activity and protein levels were close to the control values. Although the *CYP2C11* mRNA level was elevated, it was significantly lower than after the lowest dose of the antagonist and was accompanied by subtle alterations in pituitary somatostatin and a serum GH concentration. Thus, it seems that a negative hormone feedback and posttranscriptional mechanisms may be involved in the discrepancies observed between the enzyme expression, and activity, and GH levels. GH also contributes to a positive regulation of other CYP enzymes including CYP3A.

There is substantial evidence supporting the notion that glutamate significantly influences the stress responses of the hypothalamo–pituitary–adrenal (HPA) axis by acting through postsynaptic ionotropic glutamate receptors, including NMDA and non-NMDA receptors, which are localized on neurons within the hypothalamic paraventricular nuclei [22]. In line with these data, our experimental findings revealed a reduction in the serum corticosterone levels following a 5-day intracerebral administration of the selective NMDA receptor, the GluN2B subunit antagonist. The expressions of the CYP2C6 and CYP2D enzymes (studied as protein levels), which are not susceptible to hormonal regulation, were not changed by the antagonist.

The observed differences in the effects evoked by the lowest and highest doses of the antagonist may arise from its distribution within the periventricular organs involved in the neuroendocrine regulation of cytochrome P450. Considering the site of drug injection (brain lateral ventricles), the anatomic positions of the hypothalamic nuclei and pharmacokinetic aspects, it is conceivable that the lowest dose of the antagonist had a greater effect on the paraventricular nuclei releasing somatostatin and corticoliberin than on the arcuate nuclei secreting GHRH, while the highest dose acted with a similar potency on both hypothalamic nuclei, because of its wider distribution within circumventricular brain areas.

Based on our previous and present investigations involving the intraperitoneal and intracerebral administration of CP-101,606, respectively, wherein we noted changes in the growth hormone and corticosterone levels, we postulate that the brain NMDA receptor is involved in the neuroendocrine regulation of cytochrome P450 enzymes in the liver. However, the effect of this regulation is dependent on the way that drug administration and its distribution within the brain areas involved in the synthesis and secretion of hormones that positively or negatively regulated CYP enzymes (Table 1). Particularly noteworthy are the opposite effects of CP-101,606 on the GH secretion and CYP2C11 expression/activity observed after its intraperitoneal and intracerebral administration, i.e., a decrease and an increase, respectively.

Furthermore, besides the presence of glutamatergic receptors on parvocellular hypothalamic neurons, which produce releasing or inhibiting hormones, there are potential interactions between the glutamatergic system with other neurotransmitter systems involved in the pituitary hormone secretion, such as dopaminergic, noradrenergic, or serotonergic systems [39,40,41,42,43,44], which play a significant role in the regulation of hypothalamic and pituitary hormone secretion and liver cytochrome P450 expression [8,9,10]. Therefore, it cannot be excluded that those interactions also contribute to the observed effects of the NMDA receptor antagonist on the neuroendocrine regulation of physiological processes including cytochrome P450-mediated metabolism.

It is conceivable that the brain noradrenergic and serotonergic systems possessing their neuronal bodies in the brainstem may be affected by the glutaminergic system to different extents after the intraperitoneal and intracerebroventricular administration of CP-101,606 [45,46,47]. These deserve special attention since the medullary neuronal groups A1 and A2 (containing noradrenaline), which innervate the ARC and positively regulate GHRH-GH secretion, are located far from the place of intracerebroventricular injection, and thus might not be easily reached by CP-101,606 in our present work [8,46], but they were affected by the antagonist after its intraperitoneal administration in our previous study [33]. The limitation of our present study is the lack of measurement of CP-101,606 distribution and NMDA receptor occupancy within the brain after the intraventricular administration of the drug. Therefore, further studies with the local injection of CP-101,606 to the hypothalamic nuclei PVN and ARC or to the brain areas containing noradrenergic (A6—the locus coeruleus, A1 or A2) or serotonergic (dorsal and median raphe nuclei) cell bodies are advisable to fully explain the mechanisms of the central neuroendocrine regulation of the liver cytochrome P450 by NMDA receptors.

## 4. Materials and Methods

### 4.1. Animals

This study utilized adult Wistar Han rats sourced from Charles River Laboratories (Sulzfeld, Germany). Male rats weighing 225–250 g were housed under standard laboratory conditions, maintaining a temperature of 22 ± 2 °C, a relative humidity of 55 ± 5%, and a 12:12 h light/dark cycle. They were given free access to food and water. All experimental procedures followed the regulations outlined in the 86/609 EEC Directive and the Guide for the Care and Use of Laboratory Animals. This study was conducted with approval from the Local Bioethics Committee at the Maj Institute of Pharmacology, Polish Academy of Sciences (Kraków, Poland).

### 4.2. Drugs and Chemicals

The specific antagonist CP-101,606 (Traxoprodil mesylate), which selectively inhibits the GluN2B subunit of the NMDA receptor, was procured from Axon Medchem (Groningen, The Netherlands). ketamine hydrochloride (Biowet, Puławy, Poland) and Sedazin (xylazine hydrochloride, Biowet, Puławy, Poland) were used for anesthesia. The necessary reagents for assessing the enzymatic activity of CYP enzymes (including glucose-6-phosphate-dehydrogenase, glucose-6-phosphate, NADP, and NADPH) and 1′-hydroxybufuralol, were obtained from Sigma (St. Louis, MO, USA). Bufuralol synthesis was carried out at the Maj Institute of Pharmacology, Polish Academy of Sciences. Specific substrates and their corresponding metabolites were employed to determine the activity of respective CYP enzymes: caffeine, 1,3,7-trimethyluric acid and paraxanthine from Sigma (St. Louis, MO, USA), testosterone, and its hydroxymetabolites from Steraloids (Newport, KY, USA), warfarin from Merck (Darmstadt, Germany), and 7-hydroxywarfarin synthesized at the Maj Institute of Pharmacology, Polish Academy of Sciences [33]. All the organic solvents, including those of HPLC grade, were purchased from Merck (Darmstadt, Germany). The primary polyclonal antibodies, including anti-rat CYP1A1/2 from Millipore (Temecula, CA, USA), anti-rat CYP2A, CYP2D6, and CYP3A4 from the Fine Test (Wuhan, China), and the anti-rat CYP2C11 from Thermo Fisher Scientific (Walthman, MA, USA), as well as the monoclonal mouse antibodies targeting rat CYP2B1/2B2, CYP2C6 and anti-rat β-actin from Santa Cruz (Dallas, TX, USA) were utilized. Horseradish peroxidase-labeled secondary antibodies, including goat anti-mouse antibodies from Jackson ImmunoResearch (West Grove, PA, USA) and goat anti-rabbit antibodies from Vector Laboratories (Burlingame, CA, USA) were employed. The rat cDNA-expressed Supersomes, encompassing CYP1A1, CYP1A2, CYP2B1, CYP2C6, CYP2C11, CYP2D6, CYP3A1, and CYP3A2, were sourced from Gentest Corp. (Woburn, MA, USA). The SignalBoostTM Immunoreaction Enhancer Kit from Millipore (Burlington, MA, USA) was utilized for antibody dilution. The chemiluminescence reagents, specifically the LumiGlo kit, were obtained from KPL (Gaithersburg, MD, USA). To isolate RNA, the Total RNA Mini kit provided by A&A Biotechnology (Gdynia, Poland) was utilized. The High-Capacity cDNA Reverse Transcription Kit, TaqMan assay, and TaqMan Gene Expression Master Mix were delivered by Life Technologies (Carlsbad, CA, USA). The ELISA kits provided by the Bioassay Technology Laboratory (Shanghai, China) were employed to measure the levels of serum growth hormone (GH), corticosterone, T_3_, T_4_, and pituitary somatostatin. Additionally, the ELISA kit from Elabscience Biotechnology Co., Ltd. (Bethesda, MD, USA) was used specifically for detecting pituitary growth hormone-releasing hormone (GHRH).

### 4.3. Animal Treatment and the Preparation of Microsomes

The bilateral implantation of guide cannulas in the lateral brain ventricles was performed under anesthesia (ketamine HCl 65 mg/kg i.p. and xylazine HCl 5 mg/kg i.p.) as described earlier [9]. One week after inserting the cannulas in the brain, CP-101,606 was injected into the lateral ventricles once a day for 5 days without any anesthetic agents. The animals received CP-101,606 at doses of 6, 15, or 30 µg/brain (3, 7.5, or 15 µg in 5 µL of 0.9% NaCl per ventricle) or solvent in the control group (5 µL of 0.9% NaCl per ventricle) once a day for a duration of 5 days (the given doses refer to pure CP-101,606). The experimental groups consisted of 10 rats each, including a control group and CP-101,606-treated groups. Following the completion of treatment, the rats were sacrificed via decapitation 2 h after the last dose. Pituitaries and livers were promptly frozen in dry ice and stored at −80 °C for subsequent analysis. Blood serum was obtained through centrifugation and stored at −80 °C. The liver microsomes were prepared using standard conditions involving homogenization in a 20 mM Tris/KCl buffer at pH 7.4, followed by washing with 0.15 M KCl.

### 4.4. Assessment of Cytochrome P450 Enzyme Activities in Microsomes Isolated from the Liver

The enzymatic activities of CYP enzymes in liver microsomes were investigated through CYP-specific reactions performed under previously optimized conditions [9,33,48]. In brief, the activity of CYP1A was determined by measuring the rate of caffeine C-8-hydroxylation and 3-N-demethylation. The activity of CYP2C6 was assessed by monitoring the rate of warfarin 7-hydroxylation. CYP2D activity was quantified by measuring the rate of bufuralol 1′-hydroxylation. The activities of CYP2A, CYP2B, CYP2C11, and CYP3A were estimated by measuring the rates of testosterone hydroxylation at positions 7α, 16β, 2α and 16α, and 2β and 6β, respectively. The resulting metabolites generated from specific substrates were quantified using high-performance liquid chromatography (HPLC) with UV or fluorescence detection. CYP activity results were obtained from groups of 9–10 rats in both the control group and the groups treated with CP-101,606.

### 4.5. Assessment of CYP Protein Levels in the Liver Microsomes

Western blot analysis was conducted following previously established protocols [9,33]. In summary, microsomal proteins (10 µg for CYP1A, 2A, and 5 µg for CYP2B, 2C6, 2C11, 2D, 3A) were loaded onto SDS gels in a Laemmli buffer system and subsequently transferred to a nitrocellulose membrane. The blots were probed with primary antibodies (polyclonal rabbit anti-rat CYP1A, 2A, 2C11, 2D6, and monoclonal mouse anti-rat CYP2B, 2C6 antibodies) and subsequently incubated with the corresponding horseradish peroxidase-conjugated secondary antibodies. The protein bands were visualized using enhanced chemiluminescence. Rat cDNA-expressed CYP enzymes (Supersomes) were utilized as standards: CYP1A1 (1 µg), 1A2 (2 µg), 2A2 (10 µg), 2B1 (5 µg), 2C6 (0.5 µg), 2C11 (1 µg), 2D6 (1 µg), 3A1 (1 µg), and 3A2 (1 µg). The immunoblots were analyzed using a luminescent image analyzer (LAS-1000) and Image Gauge 3.11 software (Fuji Film, Tokyo, Japan). β-actin immunoreactivity was employed as a normalization control. The results pertaining to CYP proteins were obtained from a total of 8 rats in both the control group and the CP-101,606-treated groups.

### 4.6. RNA Isolation and Quantitative Real-Time Polymerase Chain Reaction (qRT-PCR)

Liver RNA isolation and quantitative real-time PCR (qRT-PCR) were conducted following the established methodologies [9,33]. The frozen liver tissue was homogenized, and the total RNA was extracted using a Total RNA Mini kit. The isolated RNA was then reverse-transcribed using a Transcriptor High-Fidelity cDNA synthesis kit. Quantitative real-time PCR was carried out in duplicate using TaqMan Gene Expression Assays, TaqMan Expression Master Mix, species-specific TaqMan probes, and primers for the selected genes: *CYP1A1* (Rn00487218_m1), *CYP1A2* (Rn1422531_m1), *CYP2B1* (Rn01457875_m1), *CYP2B2* (Rn02786833_m1), *CYP2C11* (Rn01502203_m1), and the reference gene *ACTB* (Rn00667869_m1), encoding β-actin, and with using a QuantStudio 12k Flex from Thermo Fisher Scientific (Walthman, MA, USA). Gene expression was determined using the 2-delta Ct method with the β-actin gene (*ACTB*) expression serving as the reference, as previously described [9,33]. *CYP* mRNA levels were determined based on data obtained from groups of 9–10 rats in both the control group and the groups treated with CP-101,606.

### 4.7. Assessment of Hormone Levels

Hormonal responses were evaluated two hours following the repeated administration of CP-101,606. The levels of somatostatin, GHRH, GH, corticosterone, and thyroid hormones (T_3_, T_4_) in either pituitary or serum samples were measured using ELISA kits. Absorbance readings were obtained using a Synergy Mx Monochromator-Based Multi-Mode Microplate Reader (Biotek, Winooski, VT, USA). Hormone concentrations were determined based on data collected from 8–10 rats in both the control group and the groups treated with CP-101,606.

### 4.8. Data Analysis

The presented results are expressed as the mean ± SEM and were obtained from a total of 8–10 rats in both the control group and the groups treated with CP-101,606. The statistical analysis of the alterations in hormone concentrations, liver CYP enzyme activities, protein, and mRNA levels was conducted using a one-way ANOVA followed by Tukey’s *post hoc* test. Statistical significance was considered when the *p*-value was less than 0.05 (*p* < 0.05).

## 5. Conclusions

Our study carried out the repeated intracerebroventricular injection of CP-101,606, following which a selective antagonist targeting the GluN2B subunit of NMDA receptors indicated that the brain glutamatergic system is engaged in the neuroendocrine regulation of liver cytochrome P450. The final effect of the NMDA antagonist depends on the dose and route of its administration, which determine the drug distribution within the brain and the possibility of action in different hypothalamic and nonhypothalamic areas innervated by the glutamatergic system, as well as the interactions with other neurotransmitter systems. The observed changes in liver cytochrome P450 activity evoked by the action on NMDA receptors may cause alterations in endogenous metabolism and drug biotransformation, and may contribute to drug–drug interactions.

## Figures and Tables

**Table 1:** An overview of the consequences of the 5-day intracerebroventricular injection of CP-101,606 on the expression and activity of liver cytochrome P450 enzymes and hormone secretion. A comparison with 5-day intraperitoneal administration of CP-101,606.

Route of CP-101,606 Administration	Administered Intracerebrally									Administered Intraperitoneally *		
CYP	6 µg vs. Control			30 µg vs. Control			6 µg vs. 30 µg			20 mg/kg		
	Activity	Protein Level	mRNA Level	Activity	Protein Level	mRNA Level	Activity	Protein Level	mRNA Level	Activity	Protein Level	mRNA Level
1A1/2	−	−	↑ ↑	−	↑	↑ ↑	−	−	↓ −	↓	↓	↑ ↑
2A	−	−	n.t.	−	−	n.t.	↑	−	n.t.	↓	−	−
2B1/2	(↑)	(↑)	↑ ↑	−	−	↑ −	(↑)	↑	↑ ↑	↓	−	−
2C11	↑	↑	↑	−	−	↑	↑	↑	↑	↓	↓	↓
3A	−	−	n.t.	−	−	n.t.	−	−	n.t.	↓	↓	↓
2C6	−	−	n.t.	−	−	n.t.	↑	−	n.t.	−	n.t.	n.t.
2D	−	−	n.t.	−	−	n.t.	↑	−	n.t.	↓	−	−
Hormone concentration	6 µg vs. control			30 µg vs. control			6 µg vs. 30 µg			20 mg/kg		
Triiodothyronine (T3)	−			−			−			−		
Thyroxine (T4)	−			−			−			−		
Corticosterone	↓			↓			−			↓		
Growth hormone	(↑)			(↓)			↑			↓		
Somatostatin	(↓)			(↑)			↓			n.t.		
Growth hormone- releasing hormone	−			−			−			↓		

↑ increase, ↓ decrease, (↑) tendency to increase, (↓) tendency to decrease, − no change, n.t. not tested, * based on Bromek et al. 2021 [33].

## Data Availability

Data are contained within the article.
